# Correlative Live-Cell and Super-Resolution Imaging to Link Presynaptic Molecular Organisation With Function

**DOI:** 10.3389/fnsyn.2022.830583

**Published:** 2022-02-15

**Authors:** Rachel E. Jackson, Benjamin Compans, Juan Burrone

**Affiliations:** ^1^Centre for Developmental Neurobiology, Institute of Psychiatry, Psychology and Neuroscience, King’s College London, London, United Kingdom; ^2^MRC Centre for Neurodevelopmental Disorders, Institute of Psychiatry, Psychology and Neuroscience, King’s College London, London, United Kingdom

**Keywords:** synapse, super-resolution imaging, neurotransmitter release, calcium, active zone (AZ), correlative imaging

## Abstract

Information transfer at synapses occurs when vesicles fuse with the plasma membrane to release neurotransmitters, which then bind to receptors at the postsynaptic membrane. The process of neurotransmitter release varies dramatically between different synapses, but little is known about how this heterogeneity emerges. The development of super-resolution microscopy has revealed that synaptic proteins are precisely organised within and between the two parts of the synapse and that this precise spatiotemporal organisation fine-tunes neurotransmission. However, it remains unclear if variability in release probability could be attributed to the nanoscale organisation of one or several proteins of the release machinery. To begin to address this question, we have developed a pipeline for correlative functional and super-resolution microscopy, taking advantage of recent technological advancements enabling multicolour imaging. Here we demonstrate the combination of live imaging of SypHy-RGECO, a unique dual reporter that simultaneously measures presynaptic calcium influx and neurotransmitter release, with *post hoc* immunolabelling and multicolour single molecule localisation microscopy, to investigate the structure-function relationship at individual presynaptic boutons.

## Introduction

Synapses are remarkably heterogenous subcellular compartments, formed of a presynaptic terminal and a postsynaptic element that vary in structure, function and molecular composition. At the presynaptic terminal, calcium influx through voltage gated calcium channels (VGCCs) triggers the fusion of a neurotransmitter-filled synaptic vesicle (SV) with the plasma membrane at the active zone (AZ), a specialised site containing the necessary machinery for vesicle tethering, docking and release (Südhof, 2012). Individual synapses display a high degree of heterogeneity in all stages of this process, differing in their expression of calcium channel subtypes (Luebke et al., 1993; Wheeler et al., 1994; Reid et al., 1997; Gasparini et al., 2001), the level of calcium influx (Ermolyuk et al., 2012) and the sensitivity of release to calcium (Ermolyuk et al., 2012; Jackson and Burrone, 2016; Rebola et al., 2019). Most importantly, neurotransmitter release is a probabilistic process, with SV fusion occurring with a release probability (P_*r*_) that varies widely between synapses (Murthy et al., 1997). Presynaptic boutons are also heterogenous in their structure, such as volume and shape, as well as in their sub-synaptic features, such as AZ area and the number of neurotransmitter-filled vesicles, including their distribution in different vesicle pools (Schikorski and Stevens, 1997; Holderith et al., 2012). Although several of these aspects of presynaptic structure correlate with functional measures, our understanding of this important relationship remains incomplete, in part due to the technical challenges associated with studying both structure and function at the level of the individual synapse. The small size of presynaptic structures, below the spatial resolution limit of light microscopy, requires the use of electron or super-resolution microscopy, whilst the small and rapid events associated with neurotransmitter release require highly sensitive probes with good temporal resolution. It is therefore necessary to combine imaging modalities to uncover how structure informs function at the chemical synapse.

### Methods to Study Synapse Function

Our fundamental understanding of presynaptic function comes from classical electrophysiological studies that identified the quantal nature of neurotransmitter release (del Castillo and Katz, 1954) and its relationship with calcium influx (Dodge and Rahamimoff, 1967). Whilst electrophysiological techniques have since been refined to enable recording from individual presynaptic terminals, including small central boutons (Novak et al., 2013), imaging techniques remain the simplest approach to study synapses in the large numbers required to understand their heterogeneity. For this, a range of chemical indicators and genetically encoded reporters have been developed to measure two key aspects of presynaptic function, calcium influx and neurotransmitter release (reviewed in Wang et al., 2019). More recently, the development of genetically encoded voltage indicators has also allowed imaging of the shape of the AP along axons and at individual boutons (Hoppa et al., 2014; Sabater et al., 2021).

Reporters of neurotransmitter release consist of a pH-sensitive form of GFP (pHluorin), typically fused to the luminal domain of a synaptic vesicle protein such as VAMP2 (Miesenböck et al., 1998), synaptophysin (Granseth et al., 2006), or VGLUT1 (Voglmaier et al., 2006). The pHluorin fluorescence is quenched at the acidic pH inside the vesicle and undergoes a 20-fold increase upon vesicle fusion and exposure to the extracellular medium at pH7.4 (Sankaranarayanan et al., 2000). Variants of these probes now include the addition of an invariant fluorophore (tdimer2) for normalisation of the pHluorin signal (Ratio-sypHy, Rose et al., 2013) as well as red-shifted pHluorin analogues for multicolour imaging (Li and Tsien, 2012; Liu et al., 2021). More recently, fluorescent sensors that bind glutamate or GABA have been constructed (Marvin et al., 2013, 2018, 2019) to directly sense neurotransmitter release.

The most widely used genetically encoded calcium indicators are the GCaMP family (Nakai et al., 2001), which have undergone numerous iterations to improve sensitivity and kinetics. Red-shifted calcium indicators have also been produced, such as RGECO and RCaMP1, from which jRCaMP1a and jRGECO1a have been developed (reviewed in Wang et al., 2019). Many of these probes have been targeted to specific subcellular compartments, including the presynaptic terminal (Dreosti et al., 2009; Walker et al., 2013). To monitor both presynaptic calcium influx and neurotransmitter release simultaneously we constructed SypHy-RGECO (Jackson and Burrone, 2016), a dual sensor probe which also provides an optical measure of the calcium dependence of release with single synapse resolution.

This range of tools has been essential in demonstrating the heterogeneity of synapse function, but to understand the underlying structural features that govern it, they must be combined with tools that provide information on synapse morphology and molecular organisation.

### Methods to Study Synapse Structure

Electron microscopy (EM) provided the first insights into synapse structure, revealing the existence and arrangement of SVs within a presynaptic terminal, the electron dense AZ with tethered vesicles poised for release and the opposing postsynaptic density (PSD) (Harris and Weinberg, 2012), now known to precisely cluster neurotransmitter receptors (Nair et al., 2013). Although unparalleled in its spatial resolution, EM does not provide information on the distribution of molecules unless combined with immunogold labelling which remains challenging to achieve with high labelling efficiency. Cryo-electron tomography holds great promise to study the structure of the synapse and its proteins in their native state but remains a specialised and experimentally demanding technique.

Over the last two decades, super-resolution microscopy (SRM) has emerged as a tool that allows examination of protein organisation at the nanoscale using standard fluorescent labelling approaches (reviewed in Sigal et al., 2018). Whilst it does not reach the spatial resolution of EM, it is a vast improvement over conventional light microscopy, provides information on the nanoscale organisation of molecules and is more amenable to higher throughput imaging. SRM has shown that several key AZ proteins such as Bassoon (Dani et al., 2010; Glebov et al., 2017), RIM1/2 (Tang et al., 2016; Haas et al., 2018) and Munc13 (Sakamoto et al., 2018) are arranged into subsynaptic domains (SSDs), as are postsynaptic proteins including PSD95 (Fukata et al., 2013; MacGillavry et al., 2013; Nair et al., 2013) and AMPA receptors (Nair et al., 2013). VGCCS have been shown to be highly dynamic at the presynaptic membrane, but transiently stabilise into SSDs at the AZ through protein interactions (Schneider et al., 2015; Heck et al., 2019). Importantly, SRM has brought the pre- and post-synaptic compartments together at the nanoscale by uncovering a novel arrangement in which RIM1/2 and PSD-95 SSDs align across the synaptic cleft, creating a transsynaptic nanocolumn for the efficient transfer of information across the cleft (Tang et al., 2016; Haas et al., 2018). In an intriguing twist to this arrangement, the nanoscale organisation of synaptic proteins was also shown to be plastic, suggesting an important role of subsynaptic domains in regulating synaptic function (Tang et al., 2016; Glebov et al., 2017; Hruska et al., 2018; Heck et al., 2019; Compans et al., 2021). However, the link between nanoscale organisation of synaptic molecules and synapse function is only just beginning to be understood.

### Studying the Structure-Function Relationship at the Synapse

Correlative light and electron microscopy studies have revealed that release probability is tightly coupled to the number of docked vesicles, which constitutes the readily releasable pool (RRP) (Murthy et al., 2001; Branco et al., 2010), and that both properties correlate with area of the AZ (Holderith et al., 2012). Whilst these correlations are maintained within synapses from a single type of neuron, synapses from different neuron types but with similar ultrastructural features, such as number of docked vesicles, display different release probabilities (Xu-Friedman et al., 2001).

Neurotransmitter release is driven by calcium influx such that the average release probability of a vesicle in the RRP is correlated with the size of the calcium transient (Ermolyuk et al., 2012). The number of VGCCs also scales linearly with AZ area and release probability in some synapse types (Holderith et al., 2012; Miki et al., 2017). However, weak cerebellar granule cell synapses express greater numbers of Cav2.1 channels than the stronger stellate cell synapses, but these channels are located at a greater distance from SV release sites and are therefore less effective at initiating neurotransmitter release (Rebola et al., 2019). This relationship can be further complicated by the expression of different calcium channel subtypes (Wheeler et al., 1994; Reid et al., 1997; Gasparini et al., 2001) and splice isoforms (reviewed in Lipscombe et al., 2013) that display different dynamics at the plasma membrane as well as distinct biophysical properties, which affect P_*r*_ (Heck et al., 2019). In addition, molecular crowding may affect the recruitment and retention of calcium channels in the plasma membrane. For example, blockade of neuronal activity causes a decrease in the density of Bassoon clusters at the active zone and the preferential recruitment of Cav2.1 to synapses with lower density (Glebov et al., 2017). Taken together it is clear that both the number of synaptic proteins and their spatial relationships are important in determining synaptic function.

A key spatial relationship at the synapse is the alignment of pre and postsynaptic protein clusters across the synaptic cleft in transsynaptic nanocolumns, which define the SSDs at which neurotransmitter release will maximally activate postsynaptic receptors (Nair et al., 2013; Tang et al., 2016; Haas et al., 2018). Until recently it was thought that the AZ constituted a single release site, however precise localisation of vesicle fusion using vGlut-pHluorin has revealed that there are multiple hotspots for release at each AZ, ranging from 5 to 14 (Maschi and Klyachko, 2017). Combining this with live SRM has shown that these hotspots are marked by dense nanoclusters of RIM1/2 (Tang et al., 2016), a protein whose levels at the AZ have been intimately linked to P_*r*_ (Holderith et al., 2012), and constitute distinct release sites. Munc13-1 and syntaxin-1 are similarly organised into SSDs, and their numbers also correlate with the number of release sites as well as the number of vesicles in the RRP (Sakamoto et al., 2018). However, in contrast to previous studies (Branco et al., 2010; Holderith et al., 2012), the number of Munc13-1 molecules, and consequently the number of release sites and RRP size, did not correlate with P_*r*_ at individual synapses (Sakamoto et al., 2018), suggesting that our understanding of the relationship between the molecular composition of release sites and release probability remains incomplete.

Many studies relating synaptic nanoscale structure with function have done so using indirect methods (Glebov et al., 2017) and only a few have attempted to combine SRM techniques and functional imaging at the same synapses (Tang et al., 2016; Sakamoto et al., 2018; Holderith et al., 2020), despite the insight this type of correlation can provide. Here we present a correlative approach combining live imaging of SypHy-RGECO, a genetically encoded reporter that simultaneously measures calcium influx and neurotransmitter release (Jackson and Burrone, 2016), with multicolour 3D-dSTORM using spectral demixing (Testa et al., 2010; Platonova et al., 2015). Using the well-characterised presynaptic proteins Bassoon and Cav2.1, we provide proof of principle that both nanoscale organisation and functional measures can be investigated at the level of individual boutons.

## Materials and Methods

**FIGURE 1:**
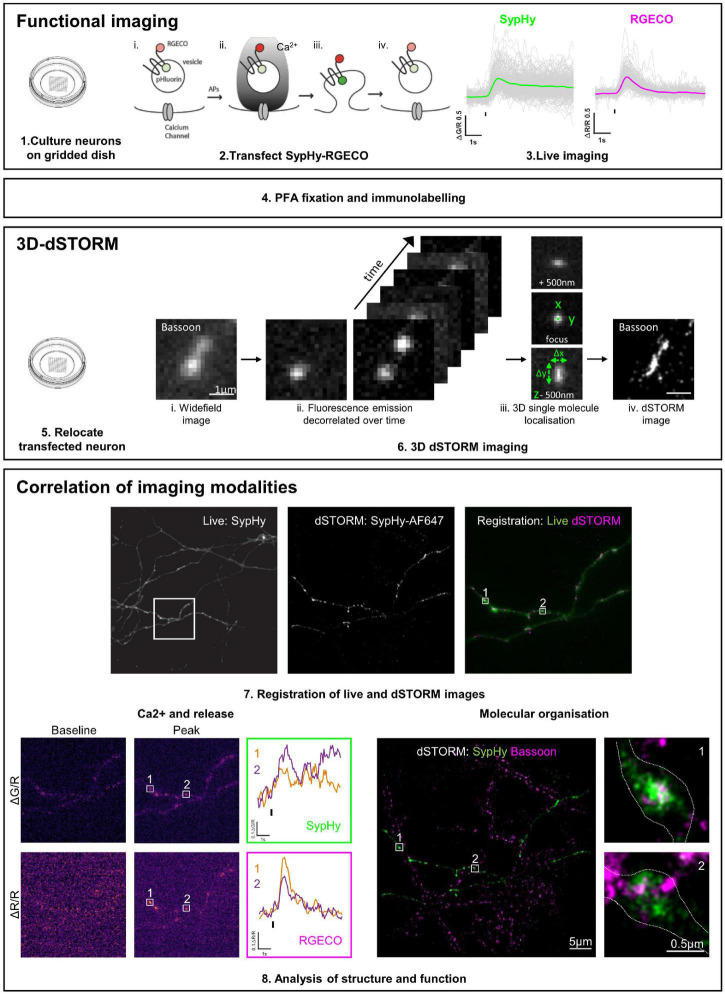
Functional and 3D-dSTORM correlative imaging pipeline. (1–3) Preparation of neurons for functional imaging of a presynaptic calcium and vesicle release sensor (SypHy-RGECO). (4) Fixation and immunolabelling of neurons. (5–6) Relocation and super-resolution (dSTORM) imaging of SypHy-RGECO + ve neurons. (7–8) Registration between imaging modalities, analysis of calcium influx (RGECO) and neurotransmitter release (SypHy) and nanoscale organisation of synaptic proteins (dSTORM), followed by single synapse correlation between modalities.

An overview of the pipeline for functional and 3D-dSTORM correlative imaging is shown in Figure 1.

### Step 1: Preparation of Hippocampal Neurons on Gridded Dishes

Hippocampi were dissected from E18.5 Wistar rat embryos (Charles River Laboratories, United Kingdom), treated with trypsin (Worthington, United Kingdom) at 0.5 mg/ml and triturated with fire-polished Pasteur pipettes. Dissociated cells (80–100k) were plated onto 35 mm low profile Grid500 μ-dishes (Ibidi, Germany) pre-treated with 100 μg/mL poly-D-lysine (Sigma, United Kingdom) and 10 μg/mL laminin (Thermo Fisher Scientific, United Kingdom). Neurons were maintained in Neurobasal media supplemented with 2% B27, 2% foetal bovine serum, 1% glutamax, and 1% penicillin/streptomycin (all Thermo Fisher Scientific, United Kingdom) in a 37°C humidified incubator with 5% CO_2_. After 3 days *in vitro* (DIV) the media was exchanged for serum and antibiotic-free media.

### Step 2: Transfection With SypHy-RGECO

At 7DIV, neurons were transfected with CAMKII:SypHy-RGECO [Jackson and Burrone, 2016, Addgene plasmid #84078, subcloned under a minimal CAMKII promoter in the vector backbone of pAAV-CW3SL-EGFP, a gift from Bong-Kiun Kaang (Choi et al., 2014), Addgene plasmid #61463] using Effectene, following the manufacturer’s protocol (QIAGEN, United Kingdom). Expression of SypHy-RGECO under the minimal CAMKII promoter should be restricted to excitatory neurons.

### Step 3: Live Imaging of SypHy-RGECO

Neurons were imaged live at DIV17-21 on an inverted Olympus IX71 microscope equipped with a 60x/1.42 NA oil objective, with a heated stage and objective to maintain the bath temperature at 32 ± 2°C. The growth media was replaced with HEPES buffered saline (HBS; 139 mM NaCl, 2.5 mM KCl, 10 mM HEPES, 10 mM D-Glucose, 2 mM CaCl_2_, 1.3 mM MgCl_2_; pH7.3 at 32°C, 290 mOsmol) supplemented with 10 μM NBQX, 25 μM APV and 10 μM Gabazine (Tocris, United Kingdom) to prevent recurrent activity. Images were acquired with an ORCA-Flash4.0 V2 C11440-22CU scientific CMOS camera (Hamamatsu Photonics, Japan) using HCImageLive Software (Hamamatsu Photonics) and the camera was cooled to approximately −20°C with an Exos2 water cooling system (Koolance, United States). The triggering of LEDs, camera acquisition and stimulation were controlled externally through Clampex software (pCLAMP10, Molecular Devices, United States) and a Master-8 programmable stimulator (A.M.P.I, Israel).

A GFP-positive cell was identified, and an extracellular parallel bipolar electrode (FHC, United States) was positioned with the tips on either side of the soma using a PatchStar Motorised Micromanipulator (Scientifica, United Kingdom). A 10-action potential (AP) stimulation at 20 Hz was delivered by an SD9 stimulator (Grass Technologies, United States) using 10 pulses of 9.5–10 V for 1 ms, a stimulus previously shown to elicit a single AP per pulse (Sabater et al., 2021). Imaging was performed with a dual band-pass filter set optimised for EGFP and mCherry (#59022, Chroma Technology, United States) and with LED excitation light sources of 470 and 585 nm (CoolLED, United Kingdom) set at 70 and 100% power, for pHluorin and RGECO fluorophores, respectively. The full frame was acquired with 4 × 4 binning (pixel size = 433 nm), alternating 20 ms exposures of the 470 and 585 nm LEDs with a 20 ms interval between frames, resulting in a final acquisition rate of 12.5 Hz for each channel.

### Step 4: Immunofluorescence

Immediately after live imaging, neurons were fixed with 4 or 1% PFA (for Cav2.1 labelling) for 10 min at room temperature (RT). Neurons were washed in PBS 3 times and 50 mM NH_4_Cl in PBS was applied as a quenching agent, followed by a further 3 washes in PBS. Dishes were stored in PBS at 4°C in the dark until staining. Neurons were permeabilised with 0.1% TritonX-100 in PBS for 10 min, washed three times in PBS and placed in blocking solution (2% BSA in PBS) for 60 min at room temperature (RT). Cells were incubated with primary antibodies diluted in blocking solution for 60 min at RT then washed three times in blocking solution for 5 min each. Secondary antibodies and nanobodies in blocking solution were applied for 60 min at RT and cells were washed three times in block and 3 times in PBS for 5 min each. Cells were kept in PBS at 4°C in the dark until STORM imaging.

### Step 5–6: Relocate Neurons for Multicolour 3D-dSTORM Imaging

dSTORM imaging was performed using a spectral-demixing SAFeRedSTORM module (Abbelight, France) mounted on an Olympus IX3 equipped with an oil-immersion objective (100 × 1.49NA oil immersion, Olympus, United Kingdom) and fibre-coupled 642 nm laser (450 mW, Errol). Fluorescent signal was collected with two ORCA-fusion sCMOS cameras (Hamamatsu). A longpass dichroic beam splitter (700 nm; Chroma Technology) was used to split the emission light on the two cameras. 3D-dSTORM imaging was performed by using cylindrical lenses placed before each camera.

The imaging buffer contained 1 mL Glucose Base (10% Glucose, 0.1% Glycerol), 125 μL of Enzyme Buffer (42 μg/mL Catalase (C100, Sigma), 1 mg/mL Glucose oxidase (G2133, Sigma), 50% Glycerol, 20 mM Tris-HCl pH7.5, 4 mM TCEP (C4706, Sigma), 25 mM KCl) and 125 μL of 1 M MEA-HCl (pH∼8 with NaOH, M6500, Sigma). This allowed a final concentration of 4 μg/mL of Catalase and 100 μg/mL of Glucose oxidase, and a final concentration of 100 mM of reducing agent MEA. The final pH was adjusted to fall between pH7.6 and pH8. To limit the contact of the imaging buffer with oxygen, the ibidi dish well was sealed with a 25 mm glass coverslip.

Image acquisition was driven by NEO software (Abbelight). The image stack contained 60,000 frames of a selected ROI of 512 × 512 pixels (pixel size = 97 nm). Cross-correlation was used to correct for lateral drifts. Super-resolution images (.tiff) with a pixel size of 10 nm and localisation files (.csv) were obtained using NEO_analysis software (Abbelight).

### Step 7: Image Registration and Synapse Correlation

A maximum z-projection of the live SypHy channel and the reconstructed dSTORM image of the amplified GFP signal were registered using landmark selection and affine transformation in Matlab (Mathworks, script available at https://github.com/jburrone/Jackson-Compans-and-Burrone). Individual synapses that were clearly defined in both images and well aligned in the registration were manually selected and included in correlative analysis.

### Step 8: Analysis of Structure and Function

#### 8a: Analysis of Live Images

Images saved in CXD format were converted to TIFF files and split into two separate channels using FIJI (ImageJ). Images were analysed using custom written Matlab scripts (Mathworks, script available upon request). Regions of interest (ROIs, 5 × 5 pixels) were selected for each punctum of SypHy fluorescence and a 15 × 15 pixel ROI was used to calculate background fluorescence. Mean background-subtracted fluorescence intensity values were calculated for each ROI in both SypHy(G) and RGECO(R) channels. Traces were smoothed by averaging over a sliding window of 4 frames. Baseline fluorescence (G_0_ and R_0_) was measured as the mean of 15 frames prior to the stimulus. ΔG and ΔR values were calculated by the change in signal intensity from the baseline, with the peak responses defined as the maximum ΔG and ΔR within 15 frames of the stimulus. Puncta in which the ΔR response to the 10AP at 20 Hz stimulus was greater than three times the standard deviation of the baseline were considered responding synapses and were included in further analysis, regardless of whether there was a measurable ΔG response. ΔG and ΔR responses were both normalised to the baseline R_0_ fluorescence, which represents the overall levels of reporter at the synapse.

#### 8b: Multicolour 3D-dSTORM Analysis

Point Clouds Analyst software (PoCA, Levet et al., 2015, 2019) was used to identify SypHy-RGECO, Cav2.1 and Bassoon clusters from localised molecule coordinates. First a Voronoi diagram was applied to draw 3D polygons of various sizes centred on the localised molecules for each colour separately. Automatic segmentation was performed to detect clusters for each protein (colour1: SypHy-RGECO to identify presynaptic boutons and colour2: Bassoon or Cav2.1). Clusters for each colour were thresholded based on their density (δ_*i*_^1^ for colour1 and δ_*i*_^2^ for colour2) with respect to the average density of the dataset for each colour, such that δ_*i*_^1^ ≥ 1δ_*d*_ and δ_*i*_^2^ ≥ 1δ_*d*_. All selected neighbouring molecules within a maximum distance between neighbours of 200 and 100 nm were considered as forming a cluster when having a minimum number of 200 and 20 molecules for colour1 and colour2, respectively. The colocalisation between SypHy-RGECO and Bassoon or Cav2.1 was computed from the overlapping clusters. We found that some colour2 clusters (Bassoon or Cav2.1) were not automatically detected as colocalising with colour 1 (SypHy-RGECO) clusters even though they clearly formed part of the same synapse. There are multiple reasons for this, including the segmentation thresholds used, which limit the extent of the overlap between colours, and the fact that SypHy-RGECO labels synaptic vesicles and does not cover the entirety of the presynaptic volume. We therefore manually verified and corrected for any missed colocalisations, then extracted cluster volume and density of molecules for colour1 and colour2 from all synapses. Synapses with no colour2 cluster or ambiguous clusters that could not be manually verified were excluded.

#### 8c: Correlation of Live and dSTORM Analysis

Functional responses (ΔG/R, ΔR/R and the ratio ΔG/ΔR) and structural parameters (number of detections and cluster volume and density) were analysed for correlation using Matlab (Mathworks, United States) and Prism 9 (GraphPad, United States), across all synapses selected during image registration (step 7) that passed the inclusion criteria in both imaging modalities (steps 8a and b).

## Results

**FIGURE 2:**
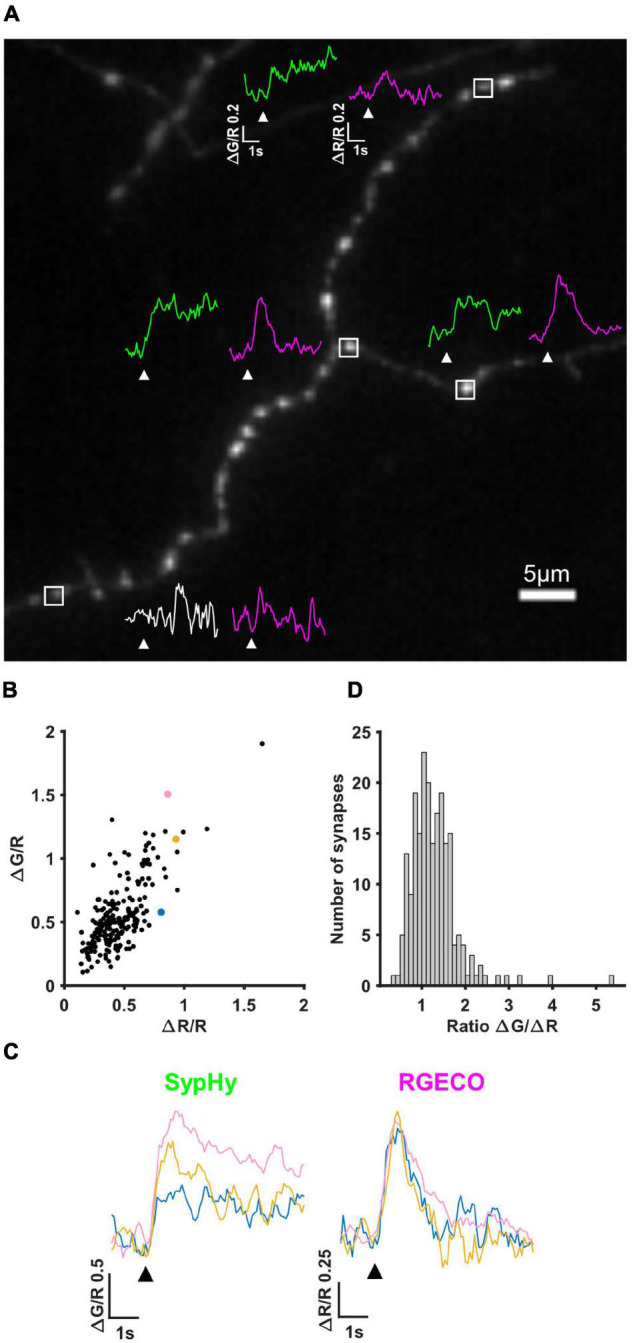
SypHy-RGECO responses to 10AP stimuli. **(A)** Image of an axon transfected with SypHy-RGECO (green channel (SypHy) shown). Representative traces of SypHy (ΔG/R, green/white) and RGECO (ΔR/R, magenta) responses to 10AP stimuli are shown for boutons with a detectable calcium influx (peak response greater than 3SD of the baseline), associated with SV release (green traces) or failure to release (bottom synapse, white trace) assessed using the same threshold. White boxes indicate selected boutons, all traces shown on the same scale, white arrows indicate stimulation. **(B)** Cells were stimulated with a 10AP 20 Hz stimulus. The change in fluorescence from baseline (ΔG and ΔR) was normalised to the basal RGECO fluorescence (R) for each synapse. ΔG/R and ΔR/R responses from all synapses show a positive correlation (Spearman’s rank correlation, *r* = 0.633, *p* = < 0.0001, *n* = 211 synapses). **(C)** SypHy (left) and RGECO (right) traces from 3 synapses (coloured points in **B**) with a similar amount of calcium influx (RGECO) in response to a 10AP stimulus but a different level of neurotransmitter release (SypHy). Black arrows indicate stimulation. **(D)** Distribution of the ratio ΔG/ΔR indicating the calcium dependence of release (for the synapses shown in **B**).

The first imaging step in our pipeline (see section “Materials and Methods” and Figure 1) is to simultaneously measure calcium influx and neurotransmitter release by performing live imaging of SypHy-RGECO, a probe that localises to presynaptic boutons, seen as clear puncta along the axons of hippocampal neurons (Figure 2A). Axonal regions were imaged whilst the soma of the transfected cell was stimulated with a parallel bipolar electrode using 10 pulses that induce single APs (Sabater et al., 2021), a stimulus which lies within the linear range for both reporters (Jackson and Burrone, 2016). Puncta in which the change in RGECO fluorescence was greater than 3S.D. of the baseline were considered functional synapses and included in later analysis (magenta traces, Figure 2A). Using the same criterion for sypHy responses, almost a quarter of synapses (23.27%) did not display measurable neurotransmitter release to a 10AP stimulus despite experiencing a calcium influx, suggesting they had a low release probability (white vs. green traces, Figure 2A). When neurotransmitter release could be quantified, individual synaptic ΔG/R and ΔR/R responses exhibited a wide range of amplitudes, which were positively correlated (Figure 2B). However, due to the spread of points within this correlation, individual synapses with the same level of calcium influx could display different levels of neurotransmitter release (Figure 2C). This can be quantified using the ratio ΔG/ΔR, a measure of the calcium-dependence of release (Figure 2D).

**FIGURE 3:**
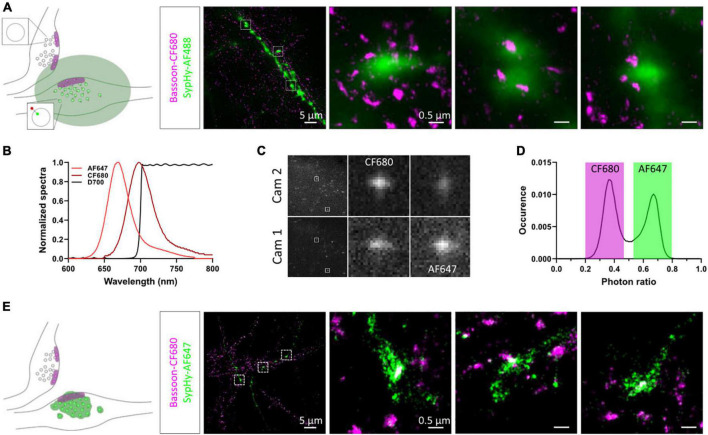
Multicolour 3D-dSTORM to investigate synaptic protein nanoscale organisation. **(A)** Left panel: schematic of two presynaptic bouton labelled for active zone protein Bassoon resolved with dSTORM. Only one of the two boutons expresses SypHy-RGECO (zoomed synaptic vesicle). The green area represents the diffracted signal from GFP staining imaged with conventional widefield fluorescence microscopy. Right panel: representative example of a widefield image of a SypHy-RGECO + ve axon immunolabelled with AF488 (green) overlaid with a dSTORM image of endogenous Bassoon (magenta) immunolabelled with CF680. Zoomed examples of 3 boutons are shown (square box). **(B)** Emission spectra of AF647 (light red) and CF680 (dark red), and transmission profile of D700 dichroic (black). **(C)** Example images of single emitters obtained from the 2 cameras on our SD-dSTORM system and zoomed examples of 2 single emitters. AF647 intensity is stronger on camera 1 than camera 2. CF680 intensity is stronger on camera 2 than camera 1. **(D)** Distribution of single emitters according to their photon ratio [I_*cam*1_/(I_*cam*2_ + I_*cam*1_)] allows separation of AF647 and CF680 detections. **(E)** Left panel: schematic of two presynaptic bouton labelled for Bassoon resolved with dSTORM. Only one of the two boutons expresses SypHy-RGECO (zoomed synaptic vesicle). The green area represents the super-resolved signal from GFP staining imaged with SD-dSTORM. Right panel: representative example of multicolour 3D-dSTORM of a SypHy-RGECO + axon immunolabelled with AF647 (green) and endogenous Bassoon (magenta) immunolabelled with CF680. Zoomed examples of 3 boutons are shown (square box).

To relocate the same synapses after fixation and staining, we used both their grid position and axonal morphology. We first amplified the SypHy signal using an anti-GFP antibody and an AF488-coupled secondary antibody and labelled endogenous Bassoon with a primary and CF680-coupled secondary antibody suitable for dSTORM imaging. SypHy-RGECO + ve boutons were identified and a widefield image was acquired in the green channel. 3D-dSTORM was then performed for CF680 and the super-resolved image of Bassoon was reconstructed and overlaid on the diffracted GFP image (Figure 3A). Due to the low resolution of the GFP image, chromatic aberrations and the high density of axons in the culture, identifying colocalisation between Bassoon subsynaptic domains (SSDs) and SypHy-RGECO + ve boutons was difficult. To overcome these limits and avoid misidentification, we took advantage of spectral demixing dSTORM (SD-dSTORM) to perform multicolour super-resolution imaging. SD-dSTORM uses fluorophores excitable with a single wavelength (here 642 nm) but with a small shift in their emission spectrum (Figure 3B). Using a long-pass dichroic to split the emission of fluorescence from single emitters onto two cameras, a photon ratio can be calculated to assign the detection to one of the fluorophores (Testa et al., 2010; Platonova et al., 2015; Figures 3C,D and Supplementary Movie 1). As SD-dSTORM uses fluorophores with similar emission wavelengths, chromatic aberrations are negligible compared to more classical multicolour SRM that requires further image processing. Using this technique increased the resolution of SypHy-RGECO + boutons and improved identification of their associated Bassoon SSDs (Figure 3E).

To further enhance the localisation precision of the SypHy-RGECO + vesicular pool, we used GFP nanobodies directly coupled with AF647. As expected, due to the reduced distance between SypHy and AF647 (Früh et al., 2021), we observed a net reduction in SypHy cluster volume with nanobody labelling compared to classical primary/secondary staining (Supplementary Figures 1A–C). However, after 1% PFA fixation required for labelling Cav2.1, nanobody staining appeared weaker and did not easily allow relocation of SypHy-RGECO + ve neurons for dSTORM imaging, correct registration of image modalities or confident identification of presynaptic boutons (Supplementary Figure 1A, top panel). When compared to nanobody staining following 4% PFA fixation, used for Bassoon labelling, the staining following 1% PFA fixation was less reliable, as shown by the decreased number of detections of AF647 per SypHy-RGECO cluster (Supplementary Figures 1A,D). Since under these conditions the GFP nanobody was likely undersampling the labelling of SypHy, we continued to use the classical primary and secondary antibody labelling approach, which appeared to be less sensitive to fixing conditions and improved the correlation between the number of SypHy-RGECO detections in dSTORM and the RGECO baseline (R_0_) in live imaging (Supplementary Figures 1E–G). Whilst the primary aim of super-resolving SypHy was to precisely identify live-imaged boutons and their associated proteins of interest, we were also able to investigate the relationship between presynaptic functional properties and SypHy-RGECO detections in neurons labelled with the polyclonal antibody. As this reporter is mostly expressed on SVs, the number and volume of the SypHy-RGECO cluster will be proportional to the total pool of vesicles. We did not observe any correlation between SV release (ΔG/R) or calcium influx (ΔR/R) and either the number or volume of SypHy detections (Supplementary Figure 2).

**FIGURE 4:**
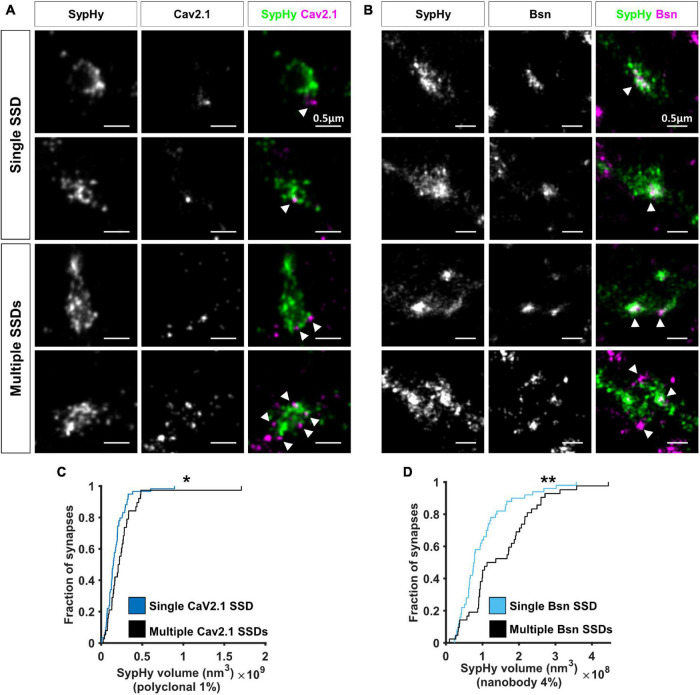
Nanoscale organisation of Cav2.1 and Bassoon in SypHy-RGECO + ve boutons. **(A,B)** Examples of SypHy-RGECO + ve boutons labelled for SypHy-AF647 (left panels) and Cav2.1-CF680 (A: middle panels) or Bassoon-CF680 (**B**, middle panels) imaged with SD-dSTORM. Merged images of SypHy (green) and Cav2.1 (**A**, magenta) or Bassoon (**B**, magenta) are shown in the right panels. White arrows indicate Cav2.1 or Bassoon subsynaptic domains (SSD). Synapses can contain a single (top panels) or multiple SSDs (lower panels). **(C)** Cumulative distribution of SypHy cluster volume labelled with polyclonal anti-GFP and AF647-coupled secondary antibodies after 1%PFA fixation, for synapses with single or multiple Cav2.1 SSDs (Kolmogorov-Smirnov test, *p* = 0.025, n_*single*_ = 59, n_*multi*_ = 38). **(D)** Cumulative distribution of SypHy cluster volume labelled with AF647-coupled anti-GFP nanobody after 4%PFA fixation, for synapses with single or multiple Bassoon SSDs (Kolmogorov-Smirnov test, *p* = 0.004, n_*single*_ = 50, n_*multi*_ = 42). *p < 0.05 and **p < 0.01.

Two colour super-resolution images of either Cav2.1 or Bassoon together with SypHy-RGECO showed that both AZ proteins are organised in sub-synaptic domains (SSDs), such that a single SypHy-RGECO cluster, representing an individual bouton, could contain anywhere from 1 to 6 Cav2.1 (mean = 1.6, Figure 4A) or Bassoon SSDs (mean = 1.7, Figure 4B). Point Clouds Analyst, a method for 3D two-colour segmentation based on single molecule detection densities (PoCA, Levet et al., 2015, 2019), was used to identify SypHy-RGECO clusters and their associated Bassoon or Cav2.1 SSDs. In most cases, the identification of Bassoon or Cav2.1 SSDs within a SypHy-RGECO cluster was straight forward (Supplementary Figure 3C). In a few cases, segmentation limits meant that associated SSDs were not automatically identified (Supplementary Figures 3D,E). Where possible, these were manually corrected, otherwise synapses were excluded from further analysis. This analysis showed that synapses with more than one Cav2.1 SSD had bigger SypHy cluster volumes (Figure 4C), a difference that was even more noticeable for synapses with multiple Bassoon SSDs (Figure 4D). Interestingly, in synapses with multiple SSDs, the individual clusters of both Cav2.1 and Bassoon display a slight but significant decrease in density compared to those from synapses with single SSDs (Supplementary Figure 4). Together these observations suggest that synapses can use multiple strategies for protein organisation such as forming one or multiple SSDs with different densities of crowding. These types of observation would not be possible using conventional fluorescence microscopy, highlighting the advantage of using SRM to investigate synaptic protein organisation.

**FIGURE 5:**
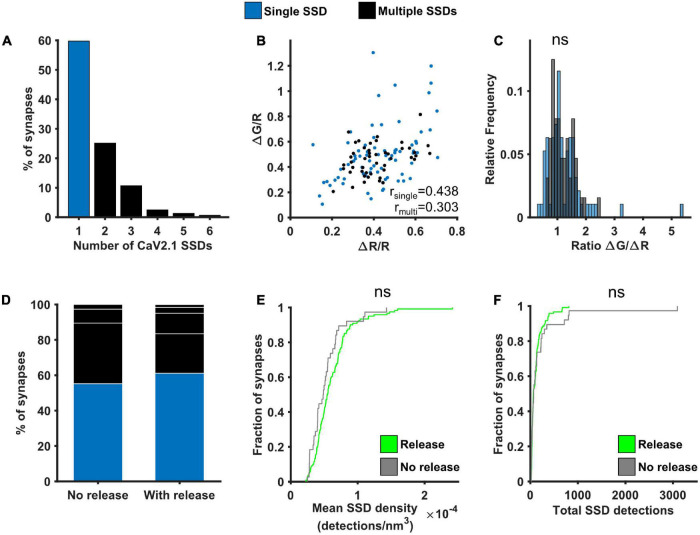
Effect of Cav2.1 nanoscale organisation on neurotransmitter release. **(A)** Distribution of the number of Cav2.1 SSDs per synapse. **(B)** ΔG/R and ΔR/R responses from synapses *post hoc* labelled for Cav2.1 and identified as containing a single (blue) or multiple Cav2.1 SSDs (black) show a positive correlation (Spearman’s rank correlation, r_*single*_ = 0.439, *p* < 0.0001, n_*single*_ = 74 synapses; r_*multi*_ = 0.303, *p* < 0.0001, n_*multi*_ = 47 synapses). **(C)** Distribution of the ratio ΔG/ΔR for the two group of synapses. Distributions are not significantly different (Kolmogorov-Smirnov test, *p* = 0.657, n_*single*_ = 74, n_*multi*_ = 47). **(D)** Percentage of synapses with single or multiple Cav2.1 SSDs in synapses without detectable release (left, *n* = 38) or with release (right, *n* = 121). White lines indicate separation of multiple SSDs by number. **(E)** Cumulative frequency distribution of the mean density of detections in Cav2.1 SSDs per synapse (number of detections/nm^3^) in synapses with (green) or without (grey) detectable release to a 10AP stimulus (Kolmogorov-Smirnov test, *p* = 0.122, n_*release*_ = 121, n_*no release*_ = 38). **(F)** Cumulative frequency distribution of the total number of detections in Cav2.1 SSDs per synapse in synapses with (green) or without (grey) detectable release to a 10AP stimulus (Kolmogorov-Smirnov test, *p* = 0.820, n_*release*_ = 121, n_*no release*_ = 38).

As VGCCs are directly involved in neurotransmitter release, we next investigated the potential link between Cav2.1 nanoscale organisation, one major subtype of VGCC found at synaptic boutons, and SV release at individual synapses. The number of Cav2.1 SSDs varied considerably across boutons, ranging from 1 to 6 SSDs, with around 60% of analysed synapses containing only a single Cav2.1 SSD (Figure 5A). We separated synapses into two groups according to the presence of a single or multiple Cav2.1 SSDs. For both groups, we observed a similar positive correlation between ΔG/R and ΔR/R (Figure 5B). Within this correlation, the spread of the responses indicates a variation in the calcium-dependence of release between synapses, quantified by the ratio ΔG/ΔR. We compared the distribution of ΔG/ΔR between synapses with single or multiple Cav2.1 SSDs and found no significant difference (Figure 5C). As we did not observe any correlation between Cav2.1 SSD properties and the level of calcium influx or neurotransmitter release (Supplementary Figure 5), we next split synapses based on their release properties—those with detectable levels of neurotransmitter release in response to a 10AP stimulus and those that do not exhibit any detectable release despite showing robust Ca^2+^ influx. No difference in the number of Cav2.1 SSDs, nor in SSD properties were observed between synapses which released and those which did not (Figures 5D–F). Our findings indicate that Cav2.1 abundance or spatial distribution are not a good predictor for measures of presynaptic function.

**FIGURE 6:**
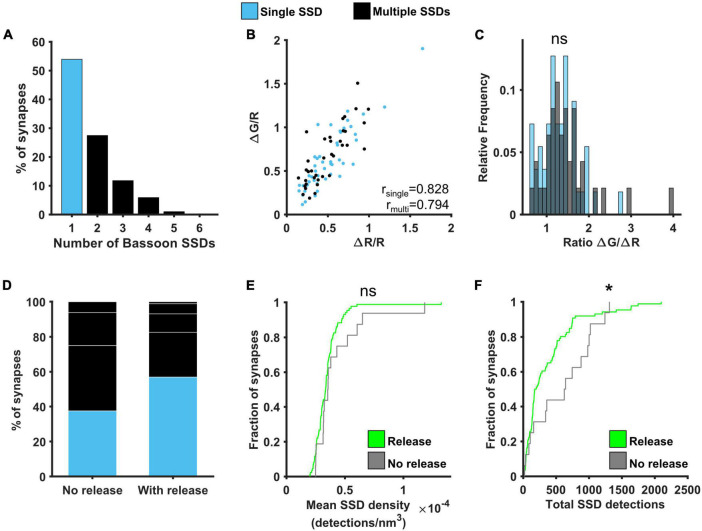
Effect of Bassoon nanoscale organisation on neurotransmitter release. **(A)** Distribution of the number of Bassoon SSDs per synapse. **(B)** ΔG/R and ΔR/R responses from synapses *post hoc* labelled for Bassoon and identified as containing a single (blue) or multiple (black) Bassoon SSDs show a positive correlation (Spearman’s rank correlation, r_*single*_ = 0.828, *p* < 0.001, n_*single*_ = 49 synapses, r_*multi*_ = 0.794, *p* < 0.001, n_*multi*_ = 37 synapses). **(C)** Distribution of the ratio ΔG/ΔR for the two group of synapses. Distributions are not significantly different (Kolmogorov-Smirnov test, *p* = 0.575, n_*single*_ = 49, n_*multiple*_ = 37). **(D)** Percentage of synapses with single or multiple Bassoon SSDs in synapses without detectable release (left, *n* = 16) or with release (right, *n* = 86). White lines indicate separation of multiple SSDs by number. **(E)** Cumulative frequency distribution of the mean density of detections in Bassoon SSDs per synapse (number of detections/nm^3^) in synapses with (green) or without (grey) detectable release to a 10AP stimulus (Kolmogorov-Smirnov test, *p* = 0.355, n_*release*_ = 86, n_*no release*_ = 16). **(F)** Cumulative frequency distribution of the total number of detections in Bassoon SSDs per synapse in synapses with (green) or without (grey) detectable release to a 10AP stimulus (Kolmogorov-Smirnov test, *p* = 0.041, n_*release*_ = 86, n_*no release*_ = 16). *p < 0.05.

The probability of neurotransmitter release was previously shown to correlate well with active zone area (Holderith et al., 2012). We therefore investigated the relationship between Bassoon, one of the main presynaptic scaffolding proteins, and presynaptic function. Around 55% of synapses contained a single Bassoon SSD, with the rest of the synapses containing from 2 to 5 SSDs, much like our observations for Cav2.1 (Figure 6A). A similar positive correlation was found between ΔG/R and ΔR/R for synapses with single and multiple Bassoon SSDs (Figure 6B) and again, there was no difference in the relationship between Ca^2+^ and release between the two group of synapses when comparing the distributions of the ratio ΔG/ΔR (Figure 6C). We also found no correlation between Bassoon SSD properties and the levels of release or calcium influx, with the exception that in synapses with multiple Bassoon SSDs, the number of Bassoon detections was negatively correlated with the calcium dependence of release (ΔG/ΔR) (Supplementary Figure 6). However, when comparing synapses with detectable levels of release to those where release is not detectable, we observed differences in the nanoscale organisation of Bassoon. Releasing synapses tended to have fewer Bassoon SSDs (Figure 6D), as well as less Bassoon detections per synapse (Figure 6F). There was, however, no difference in the mean density of Bassoon distribution (Figure 6E).

## Discussion

In this study we have described the use of a correlative approach combining functional imaging and multicolour 3D-dSTORM to investigate presynaptic structure and function at individual synapses. Using this method, we examined the nanoscale organisation of Bassoon and the VGCC Cav2.1. We found that these proteins are organised into SSDs, of which there can 1–6 per synapse (mean = 1.7 and 1.6, respectively), in agreement with previous studies using SRM (dSTORM Bassoon = 1.5, Tang et al., 2016; STED 1–4 Bassoon SSD, Hruska et al., 2018) and immunogold-EM (1–5 Cav2.1 SSD; Miki et al., 2017). In synapses with a single SSD, the density of the protein cluster was increased in comparison to the individual densities of the multiple SSDs, suggesting different strategies can be employed to arrange the same number of total proteins. At present it is not clear what determines the strategy at different synapses and further examination of the co-organisation of protein SSDs will be needed.

We found no correlation between the mean density or total number of Cav2.1 localisations and the levels of either calcium influx or neurotransmitter release. However, immunogold labelling in CA3-CA3 recurrent synapses has previously shown that the number of Cav2.1 channels correlates with AZ area, which in turn correlates with P_*r*_ (Holderith et al., 2012). In our study, the population of synapses we sampled from was more heterogeneous, since the promoter driving the expression of SypHy-RGECO could be expressed in any excitatory hippocampal neuron, which may account for any difference between the studies. Importantly, the calcium channel subtypes responsible for calcium influx have been shown to vary between synapses, adding another level of heterogeneity. Cav2.1, Cav2.2, and Cav2.3 have all been shown to support presynaptic calcium influx in glutamatergic terminals (Luebke et al., 1993; Wheeler et al., 1994; Gasparini et al., 2001), and the proportions of VGCC subtypes varied not only across hippocampal regions (Parajuli et al., 2012) but, more intriguingly, across synapses themselves, even when these synapses belong to the same neuron (Reid et al., 1997). In addition, it has been shown that postsynaptic cell identity can also influence presynaptic calcium influx and neurotransmitter release properties (Éltes et al., 2017). This heterogeneity in the complement of VGCC types, where some synapses are almost entirely reliant on one subtype, whilst others utilise a mixed population (Reid et al., 1997; Scheuber et al., 2004), needs to be better understood at the single synapse level. The complexity increases even further at the level of neurotransmitter release. In addition to VGCC number and composition, neurotransmitter release is known to be regulated by the spatial coupling of VGCCs and docked SVs (Eggermann et al., 2012; Rebola et al., 2019). The precise positioning of calcium channels next to docked vesicles is thought to be regulated by an ensemble of proteins such as RIM, ELKS and Munc13 (Wang et al., 2000; Kaeser et al., 2011; Dong et al., 2018; Quade et al., 2019) as well as the diffusive behaviour of VGCCs at the plasma membrane (Schneider et al., 2015; Heck et al., 2019). Any or all of these factors could contribute to the lack of correlation observed between Cav2.1 and functional parameters. It would be particularly interesting to use the same correlative approach to explore how the diverse complement of VGCC subtypes modulates synapse function, as well as the link between vesicle position and specific calcium channel subtypes.

Similarly, we found no correlation between the levels of Bassoon and either calcium influx or neurotransmitter release, in agreement with studies using other correlative methods (Holderith et al., 2020). We previously found that Bassoon clustering density was negatively correlated with neurotransmitter release and hypothesised this was due to a molecular crowding effect that limited the recruitment of other AZ proteins such as RIM and Cav2.1 (Glebov et al., 2017). Here, although we did not observe a negative correlation, we did find a significant increase in Bassoon detections and a larger proportion of multiple Bassoon SSDs in the small subset of synapses without detectable neurotransmitter release, consistent with increased Bassoon enrichment limiting presynaptic function. Furthermore, in synapses with multiple Bassoon SSDs in which release could be quantified, both the number and volume of Bassoon detections negatively correlated with the ratio ΔG/ΔR, a measure of the calcium dependence of release, suggesting a complex interplay between the spatial relationships of the AZ matrix and VGCCs and neurotransmitter release, which will require further investigation.

Using the number of SypHy detections obtained with dSTORM as a proportional measure of the total vesicle pool, we also saw no correlation with release or calcium influx, in agreement with EM studies showing the size of the total vesicle pool does not correlate with P_*r*_, and that bouton volume shows only a weak relationship (Branco et al., 2010; Holderith et al., 2012).

Whilst the proteins we examined in this study showed few significant correlations with presynaptic functional measures, we have provided proof of principle of a correlative approach that can be used to examine functional properties and nanoscale protein organisation at individual presynaptic boutons. Nevertheless, there are some limitations to this technique which must be considered. First, as with all types of image analysis, detection thresholds must be applied. Here, using Voronoi segmentation for dSTORM imaging, we excluded low density detections that may arise from non-specific background or from isolated molecules that are not part of a cluster, perhaps because they are diffusing. By their nature these detections would not significantly alter the total number of counts and they are unlikely to substantially contribute to synapse function since they represent a minority of detections when compared to the larger clusters of protein. For SypHy, the highest density localisations arise from the total pool of SVs, which can be localised away from the AZ (Marra et al., 2012), hence this segmentation method may exclude localisations from surface stranded protein or individual SVs docked at the AZ. As the SSDs of Bassoon and Cav2.1 are located at the AZ, in some synapses they did not overlap with the segmented cluster of SypHy and had to be manually selected as belonging to the same bouton by visually inspecting individual SypHy localisations. If there was any ambiguity as to which SSD was associated with a SypHy cluster the synapse was excluded (Supplementary Figure 3). Second, despite applying manual correction, we still found a significant number of SypHy-RGECO positive synapses without any Cav2.1 or Bassoon SSD, for which there are several possible explanations. The protein may not have been present at the bouton, which is more likely in the case of Cav2.1 as it is known that different synapses can preferentially express other Cav2 subtypes (Reid et al., 1997). This is unlikely the case for Bassoon as it is thought to be ubiquitously expressed at synapses, although boutons lacking Bassoon maintain some enrichment of VGCCs and capacity to release (Davydova et al., 2014). Another explanation could be that the protein was expressed, but at a level below our detection threshold. Whilst we used standard immunolabelling techniques with commercially conjugated antibodies that offer high efficiency labelling, this may also vary between proteins and samples. Finally, the z-range in which we can localise fluorophores is limited to ∼1 um and depending on the size and orientation of the bouton in relation to the focal plane, the SSD of Bassoon or Cav2.1 may lie outside of this range. This limitation could possibly mean that some synapses were incorrectly classified as containing a single rather than multiple SSDs. This could occur if the synapse was sufficiently large to extend beyond the 1um range or the AZ SSD was detected only at the edge of the focal range and extended beyond it, which is only likely to be the case in a small proportion of synapses.

For functional imaging we used SypHy-RGECO, due to its ability to measure two aspects of presynaptic function simultaneously. Again, thresholding must be applied for both SypHy and RGECO channels, and some synapses display a calcium response but not a measurable release. We interpret these as having low release probability, as there may be a response that cannot be distinguished from baseline noise. There is a possibility that these are silent presynaptic terminals (Crawford and Mennerick, 2012), but to determine this would require using a stronger stimulus or more sensitive indicators to improve response detection, such as iGlusNFR for neurotransmitter release (Marvin et al., 2013, 2018, 2019) and GCaMP8 (Zhang et al., 2020) or jRCaMP1a/jRGECO1a (Dana et al., 2016) for calcium influx. Our correlative approach would work equally well with any of these reporters. In addition, whilst we used a 10AP stimulation protocol, which provides a robust response within the linear range for both reporters (Jackson and Burrone, 2016), different stimulation protocols could be employed to explore synaptic function in more depth than presented here.

The correlative approach would also be applicable to link function with the organisation of other synaptic proteins of interest and could be adapted to use different SRM methods. The main drawback of dSTORM imaging is the limited number of proteins that can be imaged at once. Here we have used two-colour imaging, with one channel occupied by GFP labelling for SypHy-RGECO, which is necessary to precisely relocate the same boutons. However, SD-dSTORM could allow the addition of a third fluorophore to examine two proteins of interest alongside GFP. In the dense protein environment of the presynapse, where proteins of interest are in very close proximity, care must be taken to ensure that single molecule localisation without crosstalk is achieved. Alternatively, Exchange-PAINT, SIM or the more recently developed MINFLUX could be used to improve the multiplexing possibilities of this technique, despite their own limitations (Jungmann et al., 2014; Gwosch et al., 2020; Butkevich et al., 2021). Live-SRM or single particle tracking could also add further important information about the dynamics of the nanoscale organisation of synaptic proteins and its role in regulating the calcium-dependence of release (Heine and Holcman, 2020).

Overall, we have demonstrated a flexible method that combines two imaging modalities at the same synapse to investigate functional properties and nanoscale organisation. Large numbers of synapses can be examined using this approach, enabling the wide range of synaptic heterogeneity to be explored, which will be necessary to fully understand the synaptic structure-function link.

## Data Availability Statement

The raw data supporting the conclusions of this article will be made available by the authors, without undue reservation.

## Ethics Statement

Ethical review and approval was not required for the animal study because only schedule 1 procedures performed by a competent individual were used in these studies, which are exempt under the Animals (Scientific Procedures) Act 1986.

## Author Contributions

RJ, BC, and JB designed experiments, edited, and approved the manuscript. RJ performed and analysed functional experiments. BC performed and analysed dSTORM experiments. RJ and BC drafted the manuscript. All authors contributed to the article and approved the submitted version.

## Conflict of Interest

The authors declare that the research was conducted in the absence of any commercial or financial relationships that could be construed as a potential conflict of interest.

## Publisher’s Note

All claims expressed in this article are solely those of the authors and do not necessarily represent those of their affiliated organizations, or those of the publisher, the editors and the reviewers. Any product that may be evaluated in this article, or claim that may be made by its manufacturer, is not guaranteed or endorsed by the publisher.
