# Corrigendum: Correlative Live-Cell and Super-Resolution Imaging to Link Presynaptic Molecular Organisation With Function

**DOI:** 10.3389/fnsyn.2022.953045

**Published:** 2022-06-16

**Authors:** Rachel E. Jackson, Benjamin Compans, Juan Burrone

**Affiliations:** ^1^Centre for Developmental Neurobiology, Institute of Psychiatry, Psychology and Neuroscience, King's College London, London, United Kingdom; ^2^MRC Centre for Neurodevelopmental Disorders, Institute of Psychiatry, Psychology and Neuroscience, King's College London, London, United Kingdom

**Keywords:** synapse, super-resolution imaging, neurotransmitter release, calcium, active zone (AZ), correlative imaging

There is an error in the Funding statement. Instead of “an MSCA-IF fellowship to BC” the statement should read “this project has received funding from the European Union's Horizon 2020 research and innovation programme under the Marie Sklodowska-Curie grant agreement No 894933.”

The authors apologize for this error and state that this does not change the scientific conclusions of the article in any way. The original article has been updated.

## Publisher's Note

All claims expressed in this article are solely those of the authors and do not necessarily represent those of their affiliated organizations, or those of the publisher, the editors and the reviewers. Any product that may be evaluated in this article, or claim that may be made by its manufacturer, is not guaranteed or endorsed by the publisher.

