# Supplementary material for: Correlative Live-Cell and Super-Resolution Imaging to Link Presynaptic Molecular Organisation With Function
Source: Front Synaptic Neurosci. 2022 Feb 15;14:830583. doi: 10.3389/fnsyn.2022.830583 (PMC8885727; doi:10.3389/fnsyn.2022.830583)
Supplement: Supplementary file 1 [file Data_Sheet_1.PDF]

Supplementary Figure 1

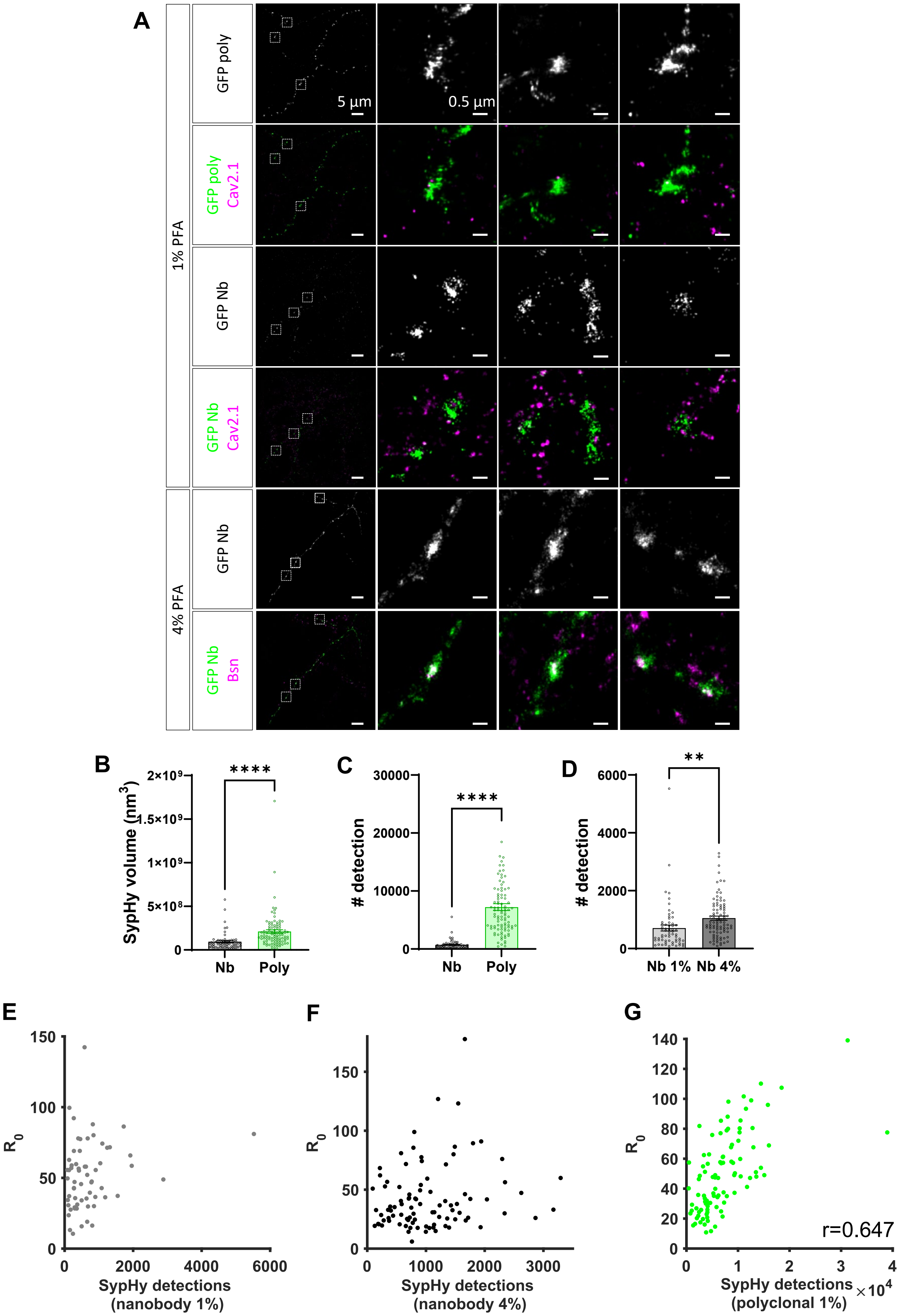

Supplementary Figure 1: Comparison of SypHy immunolabelling conditions. (A) Examples of presynaptic boutons fixed with 1% PFA and immunolabelled for GFP with either polyclonal primary and AF647-coupled secondary antibodies (top panels) or AF647-coupled nanobody (middle panels), together with CF680-Cav2.1; or fixed with 4% PFA and immunolabeled with AF647-coupled GFP nanobody and CF680-Bassoon (bottom panels). **(B)** Comparison of SypHy cluster volume (nm^3) obtained with nanobody or polyclonal antibody staining following 1% PFA fixation (unpaired t-test: $p < 0.0001$, $n_{Nb} = 62$, $n_{poly} = 97$). **(C)** Comparison of number of detections per SypHy cluster obtained with nanobody or polyclonal antibody staining following 1% PFA fixation (unpaired t-test: $p < 0.0001$, $n_{Nb} = 62$, $n_{poly} = 97$). **(D)** Comparison of number of detections per SypHy cluster obtained with nanobody staining following 1% or 4% PFA fixation (unpaired t-test: $p = 0.0057$, $n_{Nb1\%} = 62$, $n_{Nb4\%} = 92$). **(E-G)** Correlation between the total number of SypHy-AF647 detections per cluster from dSTORM images and the RGECO baseline (R_0) fluorescence intensity from live imaging, under different labelling conditions. **(E)** Nanobody labelling following 1% PFA fixation, (Spearman's rank correlation, $r = 0.24$, $p = 0.061$, $n = 62$ synapses). **(F)** Nanobody labelling following 4% PFA fixation (Spearman's rank correlation, $r = 0.169$, $p = 0.106$, $n = 92$ synapses). **(G)** Polyclonal antibody labelling following 1% PFA fixation, (Spearman's rank correlation, $r = 0.647$, $p<0.0001$, $n = 97$ synapses).

Supplementary Figure 2

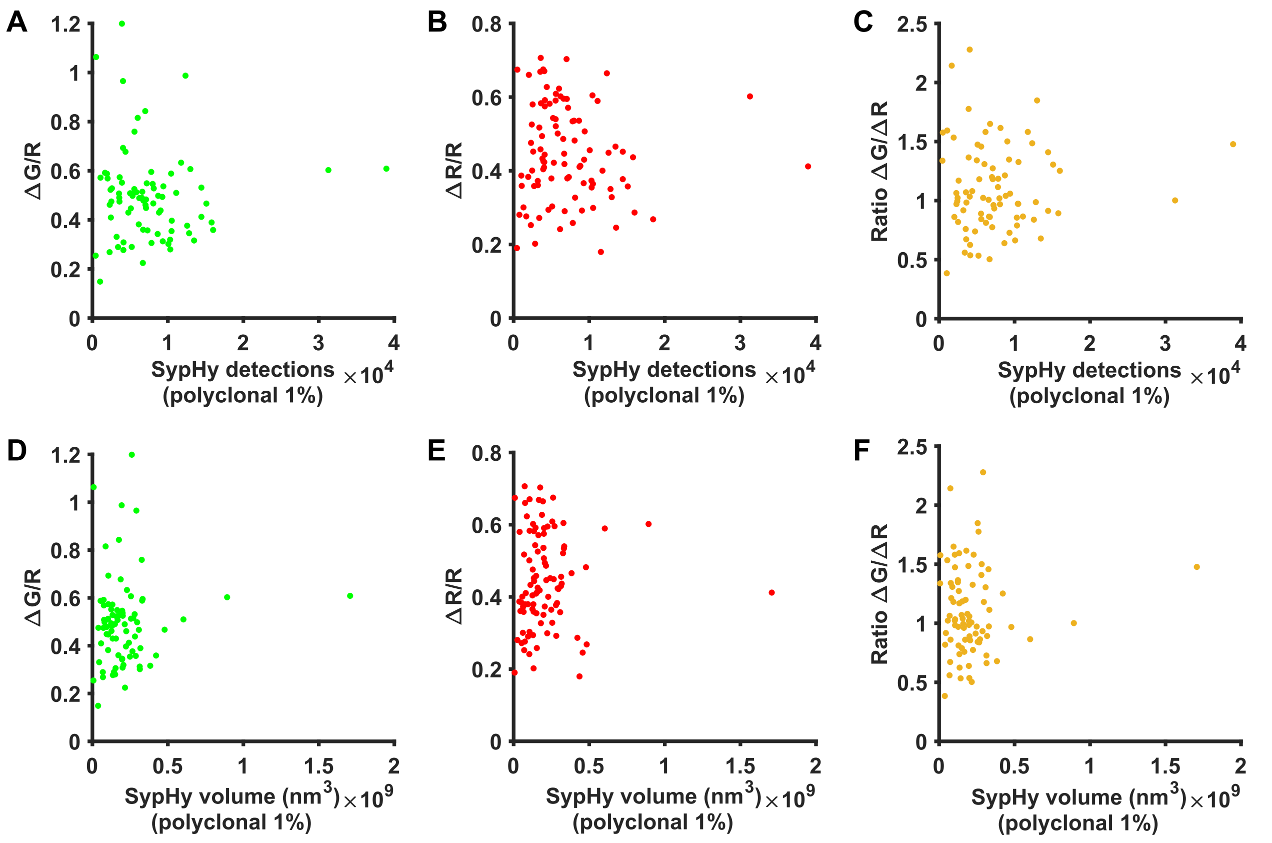

Supplementary Figure 2: Correlation between presynaptic function and SypHy cluster properties. (A-C) Correlations between the number of AF647 detections per SypHy cluster and neurotransmitter release (**A:** ΔG/R, green, Spearman's rank correlation r = 0.071, p = 0.527, n=81 synapses), calcium influx (**B:** ΔR/R, red, Spearman's rank correlation r = -0.075, p = 0.464) and calcium-dependence of release (**C:** ΔG/ ΔR, orange, Spearman's rank correlation, r = 0.013, p = 0.908). **(D-E)** Correlation between the volume of the SypHy cluster and neurotransmitter release (**D:** ΔG/R, green, Spearman's rank correlation r = 0.090, p = 0.427, n = 81 synapses), calcium influx (**E:** ΔR/R, red, Spearman's rank correlation r = 0.130, p = 0.206) and calcium-dependence of release (**F:** ΔG/ ΔR, orange, Spearman's rank correlation, r = -0.062, p = 0.584).

Supplementary Figure 3

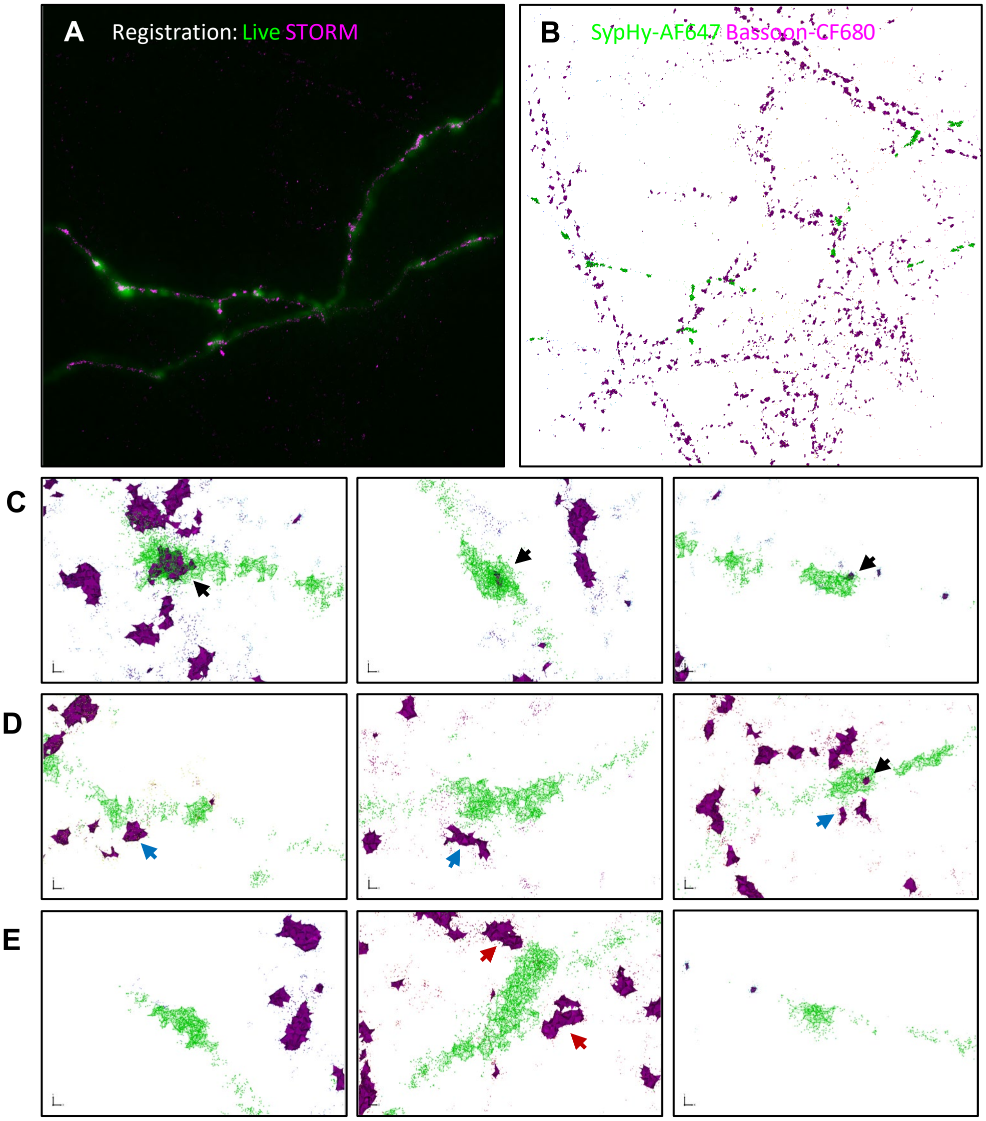

Supplementary Figure 3: Multicolour dSTORM clustering analysis. (A) Registration image from live imaging of SypHy (green) and super-resolved dSTORM image of fixed immunolabelled SypHy (magenta). **(B)** SypHy and Bassoon dSTORM image visualised using PoCA. SypHy clusters (green) and Bassoon SSDs (purple) are represented in 3D. **(C)** Zoomed images of SypHy clusters that have a clear colocalised Bassoon SSD (dark arrows). **(D)** Example SypHy synapses that do not show automatically detected colocalisation with Bassoon SSD because of cluster segmentation (blue arrows). Inspection of individual SypHy detections (green dots), that are not contained in the segmented SypHy cluster (green polygons) in 3D allowed for manual selection of these Bassoon SSDs. **(E)** Examples of SypHy synapses lacking Bassoon SSD or not showing clear evidence for colocalisation (red arrows).

Supplementary Figure 4

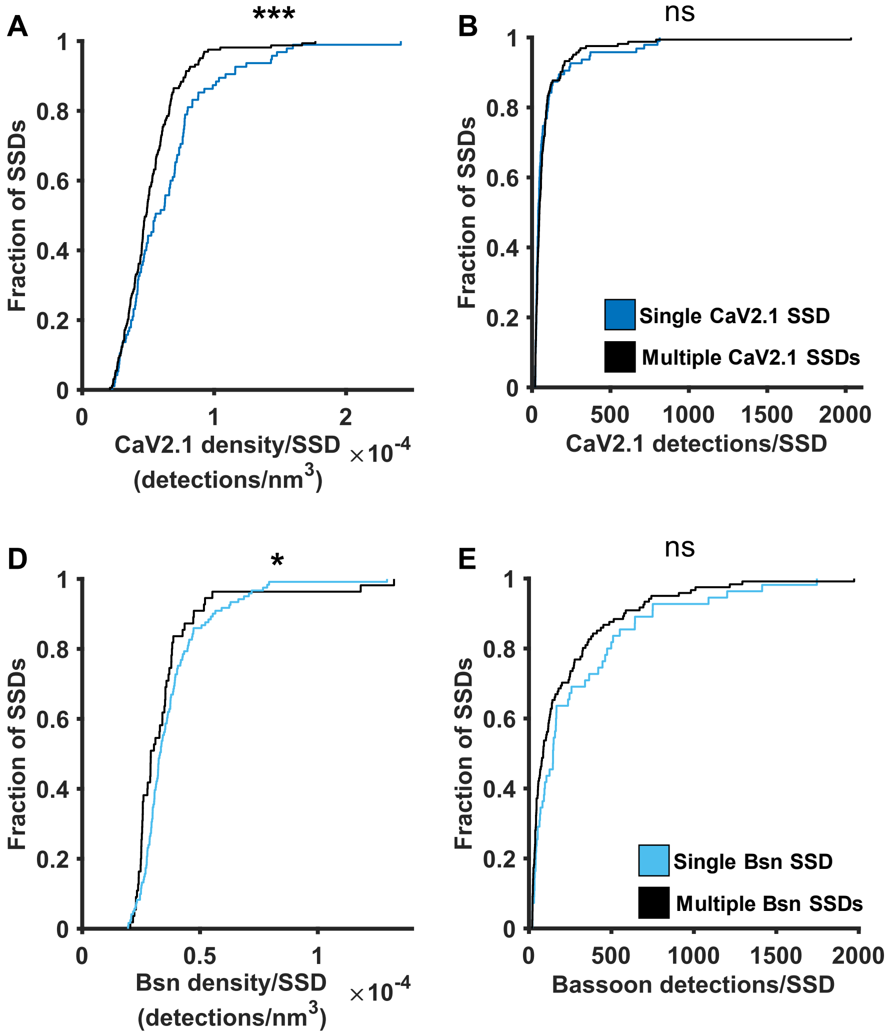

Supplementary Figure 4: Properties of single and multiple SSDs for Cav2.1 and Bassoon. **(A)** Cumulative frequency distribution of the density of CF680 detections per Cav2.1 SSD in synapses with single (dark blue) or multiple (black) Cav2.1 SSDs (Kolmogorov-Smirnov test, $p<0.001$). **(B)** Cumulative frequency distribution of the number of CF680 detections per Cav2.1 SSD in synapses with single (dark blue) or multiple (black) Cav2.1 SSDs (Kolmogorov-Smirnov test, $p = 0.430$). **(C)** Cumulative frequency distribution of the density of CF680 detections per Bassoon SSD in synapses with single (light blue) or multiple (black) Bassoon SSDs (Kolmogorov-Smirnov test, $p = 0.015$). **(D)** Cumulative frequency distribution of the number of CF680 detections per Bassoon SSD in synapses with single (light blue) or multiple (black) Bassoon SSDs (Kolmogorov-Smirnov test, $p = 0.089$).

Supplementary Figure 5

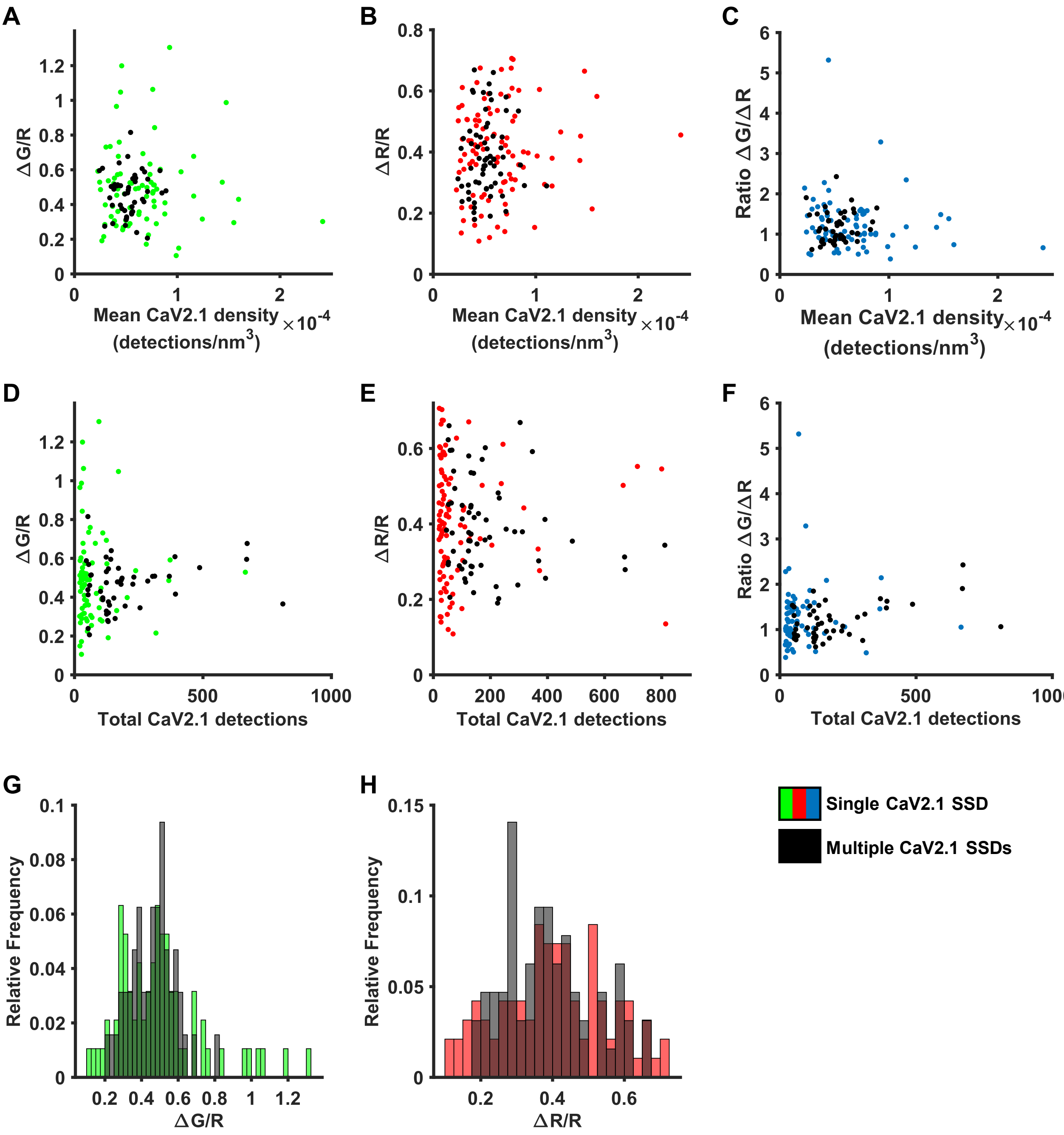

Supplementary Figure 5: Correlation between presynaptic function and Cav2.1 SSD properties. (A-C) Correlations between the mean density of CF680 detections in CaV2.1 SSDs per synapse and neurotransmitter release (**A:** ΔG/R, Spearman's rank correlation r_{single} = -0.021, p = 0.860, n_{single} = 74, r_{multi} = 0.042, p = 0.779, n_{multi} = 47), calcium influx (**B:** ΔR/R, Spearman's rank correlation r_{single} = 0.093, p = 0.370, n_{single} = 95, r_{multi} = 0.108, p = 0.396, n_{multi} = 64) and calcium-dependence of release (**C:** ΔG/ ΔR, Spearman's rank correlation, r_{single} = -0.161, p = 0.170, n_{single} = 74, r_{multi} = 0.144, p = 0.335, n_{multi} = 47). **(D-F)** Correlation between the total number of CF680 detections in CaV2.1 SSDs per synapse and neurotransmitter release (**D:** ΔG/R, Spearman's rank correlation, r_{single} = 0.011, p = 0.925, n_{single} = 74, r_{multi} = 0.160, p = 0.282, n_{multi} = 47), calcium influx (**E:** ΔR/R, r_{single} = -0.095, p = 0.361, n_{single} = 95, r_{multi} = -0.203, p =0.109, n_{multi} = 64) and calcium-dependence of release (**F:** ΔG/ ΔR, Spearman's rank correlation, r_{single} = 0.220, p = 0.060, n_{single} = 74, r_{multi} = 0.264, p = 0.073, n_{multi} = 47). Values are separated for synapses containing a single Cav2.1 SSD (green, red or blue) and multiple SSDs (black). **(G-H)** Distribution of ΔG/R **(G)** and ΔR/R **(H)** responses for synapses containing a single (green or red bars) or multiple (dark grey bars) CaV2.1 SSDs (Kolmogorov-Smirnov test **G:** p = 0.466, **H:** p = 0.244).

Supplementary Fig 6

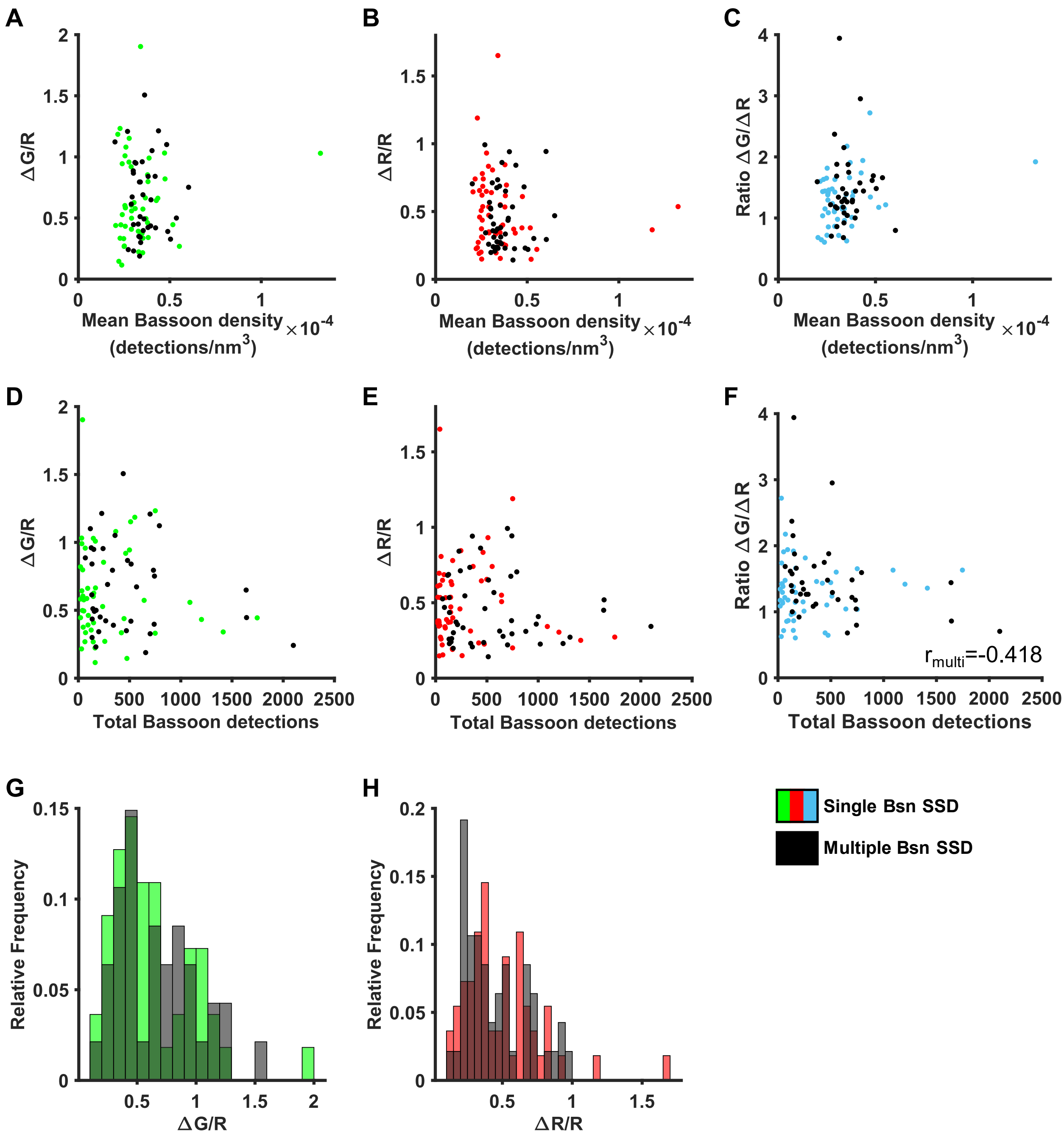

Supplementary Figure 6: Correlation between presynaptic function and Bassoon SSD properties. (A-C) Correlations between the mean density of CF680 detection in Bassoon SSDs per synapse and neurotransmitter release (**A:** ΔG/R, Spearman's rank correlation r_{single} = 0.124 p = 0.393 n_{single} = 49, r_{multi} = -0.031 p = 0.852 n_{multi} = 37), calcium influx (**B:** ΔR/R, Spearman's rank correlation r_{single} = -0.096 p = 0.484 n_{single} = 55, r_{multi} = -0.058 p = 0.70 n_{multi} = 47) and calcium-dependence of release (**C:** ΔG/ ΔR, Spearman's rank correlation r_{single} = 0.235 p = 0.104 n_{single} = 49 , r_{multi} = 0.093 p = 0584 n_{multi} = 37). **(D-F)** Correlations between the total number of CF680 detection in Bassoon SSDs per synapse and neurotransmitter release (**D:** ΔG/R, Spearman's rank correlation r_{single} = -0.061 p = 0.680 n_{single} = 49 , r_{multi} = -0.132 p = 0.435 n_{multi} = 37), calcium influx (**E:** ΔR/R, Spearman's rank correlation r_{single} = 0.017 p = 0.906 n_{single} = 55, r_{multi} = -0.037 p = 0.807 n_{multi} = 47) and calcium-dependence of release (**F:** ΔG/ ΔR, Spearman's rank correlation r_{single} = 0.026 p = 0.859 n_{single} = 49, r_{multi} = -0.417 p = 0.01 n_{multi} = 37). Values are separated for synapses containing a single Bassoon SSD (green, red or blue) and multiple SSDs (black). **(G-H)** Distribution of ΔG/R **(G)** and ΔR/R **(H)** responses for synapses containing a single (green or red bars) or multiple (dark grey bars) Bassoon SSDs (Kolmogorov-Smirnov test **G:** p = 0.459, **H:** p = 0.586).
