# Supplementary material for: Correlative Live-Cell and Super-Resolution Imaging to Link Presynaptic Molecular Organisation With Function
Source: Front Synaptic Neurosci. 2022 Feb 15;14:830583. doi: 10.3389/fnsyn.2022.830583 (PMC8885727; doi:10.3389/fnsyn.2022.830583)
Supplement: Supplementary file 2 [file Data_Sheet_2.docx]

**Supplementary Methods**

**Ethics Statement**

Only schedule 1 procedures performed by a competent individual were used in these studies, which are exempt under the Animals (Scientific Procedures) Act 1986.

**Statistical Analysis**

Synaptic functional responses (ΔG/R, ΔR/R) and dSTORM data (number and density of localisations) were tested for normality using multiple normality tests and found to be non-normal, therefore non-parametric tests were used. Correlations were assessed using Spearman’s rank correlation. Differences between distributions were assessed using the Kolmogorov-Smirnov test. N numbers report individual synapses, which are sampled from 8 cells from 5 independent cultures for Bassoon and 12 cells from 6 independent cultures for Cav2.1.

**Antibodies**

| Antibodies | Source | Identifier | Dilution |
| --- | --- | --- | --- |
| Anti-GFP Nanobody-AF647 (FluoTag-X4) | NanoTag Biotechnologies | N0304 | 1/500 |
| Chicken anti-GFP | abcam | ab13970 | 1/2000 |
| Mouse anti-Bassoon | abcam | ab82958 | 1/1000 |
| Rabbit anti-Cav2.1 | Synaptic systems | #152 103 | 1/1000 |
| Goat anti-mouse-CF680 | Biotium | #20817 | 1/1000 |
| Goat anti-rabbit-CF680 | Biotium | #20818 | 1/1000 |
| Goat anti-chicken-AF647 | Invitrogen | #A-21449 | 1/1000 |
| Goat anti-chicken-AF488 | Invitrogen | #A-11039 | 1/1000 |
